# Formulation Strategies for Enhancing Pharmaceutical and Nutraceutical Potential of Sesamol: A Natural Phenolic Bioactive

**DOI:** 10.3390/plants12051168

**Published:** 2023-03-03

**Authors:** Anroop B. Nair, Pooja Dalal, Varsha Kadian, Sunil Kumar, Minakshi Garg, Rekha Rao, Rashed M. Almuqbil, Ahmed S. Alnaim, Bandar Aldhubiab, Fatemah Alqattan

**Affiliations:** 1 Department of Pharmaceutical Sciences, College of Clinical Pharmacy, King Faisal University, Al-Ahsa 31982, Saudi Arabia; 2 Department of Pharmaceutical Sciences, Guru Jambheshwar University of Science and Technology, Hisar 125001, India; 3 Atam Institute of Pharmacy, Om Sterling Global University, Hisar 125001, India; 4 School of Pharmaceutical Sciences, Delhi Pharmaceutical Sciences and Research University, New Delhi 110017, India

**Keywords:** phytoconstituents, novel formulations, toxicity, sesamin, sesamolin, sesame oil

## Abstract

Natural plants and their products continue to be the major source of phytoconstituents in food and therapeutics. Scientific studies have evidenced the benefits of sesame oil and its bioactives in various health conditions. Various bioactives present in it include sesamin, sasamolin, sesaminol, and sesamol; among these, sesamol represents a major constituent. This bioactive is responsible for preventing various diseases including cancer, hepatic disorders, cardiac ailments, and neurological diseases. In the last decade, the application of sesamol in the management of various disorders has attracted the increasing interest of the research community. Owing to its prominent pharmacological activities, such as antioxidant, antiinflammatory, antineoplastic, and antimicrobial, sesamol has been explored for the above-mentioned disorders. However, despite the above-mentioned therapeutic potential, its clinical utility is mainly hindered owing to low solubility, stability, bioavailability, and rapid clearance issues. In this regard, numerous strategies have been explored to surpass these restrictions with the formulation of novel carrier platforms. This review aims to describe the various reports and summarize the different pharmacological activities of sesamol. Furthermore, one part of this review is devoted to formulating strategies to improve sesamol’s challenges. To resolve the issues such as the stability, low bioavailability, and high systemic clearance of sesamol, novel carrier systems have been developed to open a new avenue to utilize this bioactive as an efficient first-line treatment for various diseases.

## 1. Introduction

A large body of scientific research on bioactives has provided unquestionable assistance for the welfare of humans. Various bioactives that impart nutraceutical properties hold remarkable potential for food and pharmaceutical applications. Such bioactives have played a valuable role throughout history in the treatment and management of numerous life-threatening disorders by targeting various molecular targets, owing to their worldwide acceptance. Thus, scientific research nowadays is mainly focused on the acquisition of bioactives [1]. Numerous bioactives such as astilbin, curcumin, caffeine, silymarin, quercetin, resveratrol, citronella oil, babchi oil, eugenol, bergenin, sesamol, and carvacrol have been studied in relation to various life-threatening diseases [2,3,4,5,6,7,8,9,10]. Recently, among the various groups of natural products and plant metabolites that are available, there has been much attention given to some medicinal herbs such as sesame for their pharmaceutical applications [11].

Sesame (*Sesamum indicum* L.), which belongs to the family Pedaliaceae, is an herbaceous plant mainly cultivated for its seeds, oil, and flavorsome properties. The cognate names of this seed are til, gingelly, and benne seed, and it is commonly known as the “Queen of Oilseeds” owing to its high resistance against rancidity and oxidation [12]. Sesame is mainly cultivated in developing nations, particularly in southern latitudes. For a long period, it was chiefly used in spiritual rituals in Egypt, India, and the Persian counties.

Sesame as a potential nutraceutical resource plays a vital role in the management of various disorders owing to its high antioxidant potential. Various nutraceuticals including sesamin, sesamolin, sesamol, and sesaminol are commonly found in sesame. Among these, sesamol (SES) is the most notable and potent nutraceutical, possessing profound health benefits attributed to its free-radical-scavenging potential [13,14].

**Figure 1:**
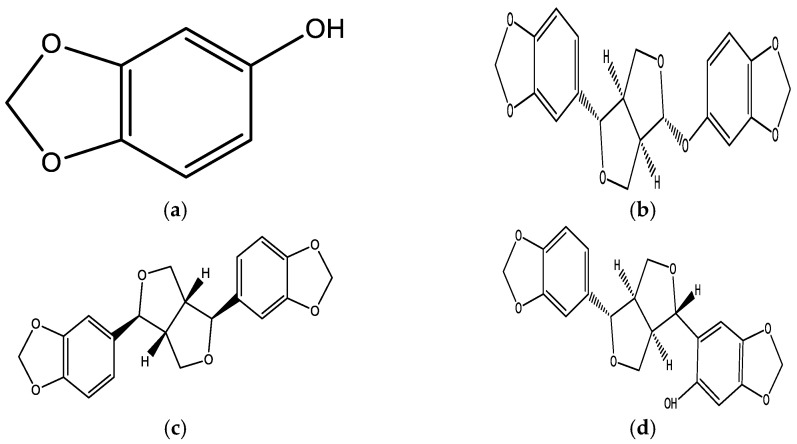
Chemical structure of (**a**) sesamol, (**b**) sesamolin, (**c**) sesamin, and (**d**) sesaminols.

Sesamol (5-hydroxy-1,3-benzodioxole or 3,4-methylene-dioxyphenol) is a phenolic lignin that is extracted from roasted sesame seeds and processed oil, which is prominently utilized in food [14]. The chemical structure of SES is shown in Figure 1. The hydroxyl group of SES is mainly responsible for its anti-inflammatory and antioxidant potential, providing it with multifunctional properties [15]. This bioactive has been found to scavenge DPPH (1,1-diphenyl-2-picrylhydrazyl) radicals, 2 imidazoquinoxaline-type radicals, and also hydroxyl radicals, superoxide anions, and singlet oxygen. It has also been evidenced in the prevention of hydroxyl-radical-induced DNA cleavage and deoxyribose degradation [15].

The growing literature of both in vivo as well as in vitro studies has emphasized various pharmacological actions of sesamol viz. hepatoprotective, anti-inflammatory, anti-atherogenic, antimutagenic, anti-cancer, and antimicrobial agent, suggesting its utility in the management of gastrointestinal, cardiovascular, metabolic, neurological, and malignant disorders, as well as its role in anti-candidal funcitons, wound healing, and aging control and management [16]. The multi-pharmacological potential of this bioactive is chiefly owing to its ability to modulate the different signaling pathways, including nuclear NF-kb (nuclear factor kappa light chain enhancer of activated B cells), AP-1 (activator protein 1), P-gp (permeability glycoprotein), MRP (material requirements planning)-1, MRP-2, glutathione, protein kinase C, ATPase, ErbB-2 (receptor tyrosine-protein kinase), AGP (Alpha-1-acid glycoprotein), COX-2 (Cyclooxygenase-2), MMPs (matrix metalloproteinases), and cyclin D1 [17]. As mentioned above, its efficacy, safety, and pharmaceutical utility are limited because of its springy solubility, poor bioavailability, rapid clearance, and low stability [18,19]. Keeping this in mind, ongoing trends of sesamol research have been engaged in finding an answer to the drawbacks associated with the conventional delivery of sesamol [18]. Taking this into account, novel delivery carriers may provide an effective solution to counter these drawbacks. Further, an effective formulation strategy leads to passively absorbed phenolic and antioxidants from the intestinal lumen to the blood and lymphatic circulation; as a result, bioavailability increases notably. Thus, researchers are experimenting on numerous new and novel carrier systems which include polymeric nanoparticles, lipid vesicles, solid lipid nanoparticles, emulsions, inclusion complexes, and nanosponges, which could open new avenues for enhancing the aforesaid restrictive factors of this herbal bioactive. Therefore, sesame and its bioactive-based novel formulations have been engineered with innovative methodologies and have been shown to evidence therapeutic potential against various health issues [20].

Considering the above-mentioned findings, an effort has been made in this review to touch upon different characteristics associated with SES-loaded novel carrier systems. Furthermore, this review also throws some light on background information for this bioactive, i.e., its mechanism of action and its therapeutic activities.

The present article summarizes the current state of SES applications in various diseases. The first part mainly represents the corresponding mechanism of sesamol in different disorders. The various therapeutic applications of this biological moiety are mentioned in the second part of this review. The last section focuses on the various novel drug carrier systems for the management of various disorders and the improvement of their drawbacks.

In this review, beneficial bio-functions of SES and their underlying mechanisms were examined and discussed. This review also highlights the various strategies to evaluate SES’s bioavailability by employing various techniques, which include nanotechnology and complexation.

## 2. Mechanism of Action

**Figure 2:**
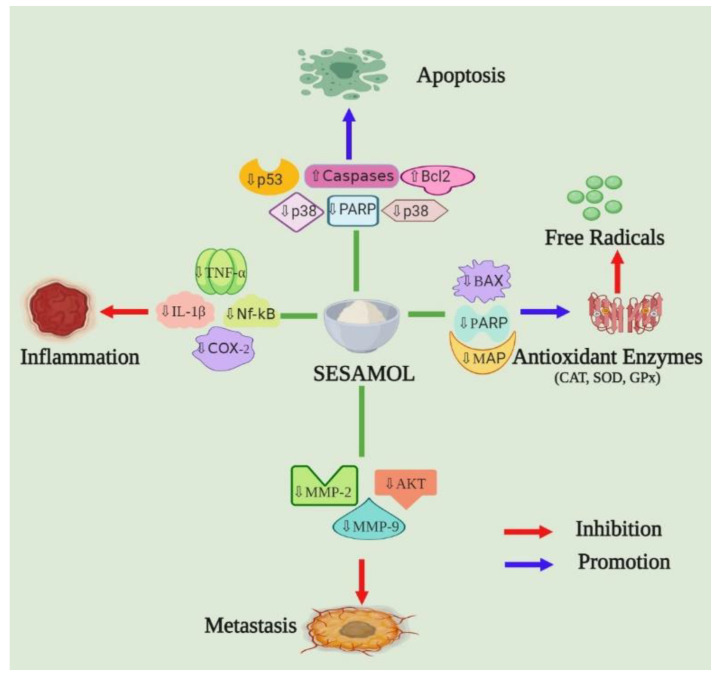
The basic mechanism of action of sesamol includes an increase in free radical scavenging, upregulation of various antioxidant enzymes, suppression of IL-1β, TNFα, LOX-1, and 5-LOX expressions, inhibition of NF-κB signaling, induction of cell apoptosis, cell cycle arrest and modulation of p53, caspase, Bcl2, and Bax expressions.

Nowadays, oxidative stress-related experimental research is dramatically growing, owing to the contribution of antioxidant mechanisms in numerous ailments including cancer, neurodegenerative diseases, cardiovascular diseases, atherosclerosis, aging, and other disorders [21]. The oxidative burden due to the excessive generation of free radicals overpowers the endogenous antioxidant potential of the body. Therefore, the overproduction of these free radicals, especially superoxide anion (O_2_•^−^ (metabolism product) and peroxyl (RO_2_•) (lipid peroxidation product) leads to protein, lipids, and DNA damage, which is directly involved in the above-mentioned diseases. On this point, the antioxidant efficiency of SES has attracted a large amount of attention from the scientific community. Previous research has demonstrated that this bioactive inhibits various radical reactions and inactivates reactive species of oxygen. The antioxidant potential depends on the redox transition, including the transfer of one hydrogen atom or electron to a radical scavenger [14,22]. This bioactive has exhibited DPPH, 2 imidazoquinoxaline radicals, hydroxyl radicals, superoxide anions, and singlet oxygen scavenging potential. During the hydrogen or electron transfer, a free radical is conveyed to the SES, creating a free radical intermediate or derivative, which is stabilized mainly by its aromatic ring resonance [22,23,24]. This bioactive has also been found effective in preventing DNA cleavage and deoxyribose degradation, induced by free hydroxyl radicals [15]. Additionally, a broad range of biological features are present in this bioactive, including lipid peroxidation inhibition, an increase in scavenging of free radicals, antioxidant enzymes upregulation, IL-1β (interleukin-1β)and TNF-α (tumor necrosis factor-α) suppression, inhibition of NF-κB (nuclear factor kappa light chain enhancer of activated B cells) and ERK (extracellular signal-regulated kinase)/p38 MAPK (mitogen-activated protein kinases) signaling, and a decrease in 5-LOX (low-density lipoprotein) and LOX-1 activity. Further, it is involved in the inhibition of PMA (phorbol 12-myristate 13-acetate)-induced MMP-1 (matrix metalloproteinases) and MMP-13 expression, initiation of tumor cells apoptosis, cell division arrest at various phases of the cell cycle, p53, caspase-3, Bcl2 and Bax expression modulation and retardation in levels of MPO (myeloperoxidase), and nitrite [14]. Collectively, all the above activities of sesamol on various pathways (shown in Figure 2) potentially allow it to be used as a therapeutic agent in various pathological conditions.

## 3. Therapeutic Potential of Sesamol

**Figure 3:**
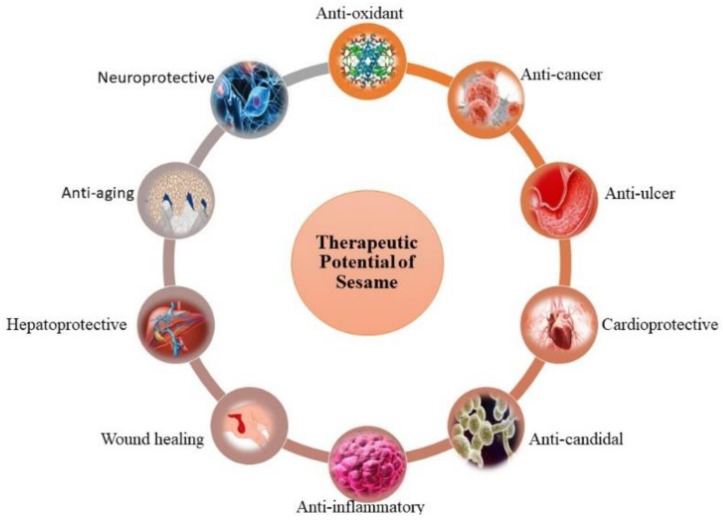
Therapeutic potential of sesame.

For a long time, the utilization of sesame oil and seeds has been associated with various health benefits. These benefits can be directly associated with the biological activities of sesame and sesamol content. Therefore, the SES bioactive is of greatest significance owing to its promising potential in the management and treatment of various ill health effects such as cardiovascular disorders, cancer, neurodegenerative diseases, inflammation, and other associated effects (Figure 3). It is a potent antioxidant and anti-inflammatory bioactive, having the capacity to modulate various pathways involved in these disorders [25].

Antioxidants from both endogenous and exogenous sources play a key role in the management of the pathogenesis of a disorder involving oxidative stress. Antioxidants or free radical scavengers can either prevent or retard oxidation or enhance the lifespan of oxidizable matter. Most disorders are generally associated with oxidative stress caused by the overproduction of free radicals. Oxidants are species that possess high reactivity and a very short half-life. These species protect various macromolecules such as DNA, proteins, and lipids, as their damage is associated with cancer, aging, ischemia, adult respiratory disorders, and rheumatoid arthritis. Antioxidants also play a vital role in any biochemical reactions that are significant in aerobic and metabolic pathways. In this way, an herbal diet shields against numerous chronic disorders associated with oxidative stress. Herbal antioxidants such as sesamol have been efficaciously utilized as rejuvenators for a long time in India and worldwide as an alternative remedy [26].

Kanimozhi and Prasad evaluated the antioxidant potency of SES and its impact on the modulation of DNA damage induced by UV radiation on Swiss albino mice. This bioactive showed antioxidant potential against hydroxyl (OH•), nitric oxide, 1,1-diphenyl-2-picrylhydrazyl (DPPH), superoxide anion (O_2_•^−^), and 2,2-azino-bis-3-ethylbenzothiazoline-6-sulphonic acid radical cation (ABTS•^+^) radicals. The outcomes of this study demonstrated that this bioactive exhibited good antioxidant potential as displayed from in vitro experiments and also advocated that SES protected DNA from damage in Swiss albino mice lymphocytes, which may be contributed by its free radical scavenging potential [27]. Yeo and his research team evaluated the antioxidant capacity of sesamol and butylated-hydroxyanisole (BHA), tert-butylated-hydroxyquinone (TBHQ), or α-tocopherol at different temperatures (90, 120, and 150 °C). The antioxidant potential of SES was found to be similar to that of TBHQ and was greater than α-tocopherol and BHA at different temperatures based on p-AV and conjugated diene hydroperoxides assays. The results of a modified DPPH assay showed that each free radical scavenger exhibited different distribution of free radical scavenging components from oxidized lipids (RSOLs) [28]. In the same year, another research group investigated marketed herbal products (olive leaf extract, sesamol, lutein, and ellagic acid) for in vitro free radical scavenging potential using the 2,2-diphenyl-1-picrylhydrazul (DPPH, ferric reducing antioxidant potential (FRAP), ABTS.+, oxygen reducing antioxidant capacity (ORAC), and β-carotene-linoleic acid assays. Antioxidant capacity was as follows: ellagic acid > sesamol > olive leaf extract > lutein for all antioxidant test methods [29]. Srisayam et al. evaluated the antimelanogenic and antioxidant skin-shielding effects of SES and positive compounds. The results showed that SES resulted in good scavenging potential for 2,2-diphenyl-1-picrylhydrazyl hydrate (DPPH^·^) radicals, with an IC_50_ value < 14.48 μM. The antioxidant power (ferric reducing antioxidant power value) of sesamol at a concentration of 0.1129 μM was observed as 189.88 ± 17.56 μM FeSO_4_ [30]. These studies advocated the strong antioxidant potential of SES and hence justified its utility in the various disorders mentioned above.

Inflammation is a complicated biological process involving numerous intermediates that are triggered by vascular tissues when tissue comes in contact with various harmful triggers such as pollens, irritants, pathogens, and damaged cells. It provides a protective effect for healing body tissues. Occasionally, inflammation events seem to turn quite severe and cause chronic diseases. Synthetic anti-inflammatory drugs are categorized as non-steroidal and steroidal anti-inflammatory agents. Further, World Health Organization (WHO) research found that none of the above-classified synthetic drugs were safer, as they are associated with many unacceptable side effects (drug-related or drug-induced) and other secondary toxic effects produced by their intake for a long duration. Hence, herbal anti-inflammatory bioactives can be positioned as a safer alternative option [31].

Chu et al. evaluated the SES potential on LPS (lipopolysaccharide), which triggered the inflammatory reactions. SES suppressed IL-1b and TNF-α, generation of nitrites in mice treated with LPS, and suppressed expression of nitric oxide synthase in mice leukocytes (RAW 264.7). By inhibiting IL-1b, TNF-α, and nitrite levels and interfering with the pathway of NF-kB, SES downregulated the LPS-initiated inflammatory response [32]. Kondamudi and his research group evaluated the effect of SES in dinitrochlorobenzene (DNCB)-induced inflammatory bowel disease (IBD) in rats. The levels of myeloperoxidase (MPO), thiobarbituric acid reactive species (TBARS), and nitrite were observed to be raised significantly (*p* < 0.05) in the rat group treated with DNCB. In contrast, a significant reduction in their levels was found in SES and sulfasalazine (SS)-treated groups. Furthermore, levels of IL-6 and TNF-α were remarkably higher in the DNCB-treated group [33]. In the following year, the same research group examined the effects of SES in the acetic-acid-induced IBD rat models. It was found that there was a significant augmentation in levels of MPO, TBARS, and nitrite in the group treated with acetic acid, whereas their levels decreased notably in SS- and SES-treated groups [34]. Periasamy et al. investigated the influence of SES on the SIR (systemic inflammatory response) in lipopolysaccharide (LPS)-induced hypotension and cecal ligation and puncture (CLP)-induced acute kidney injury in rats. This bioactive significantly suppressed creatinine, blood urea nitrogen9BUN), IL-6, IL-1β, and nitrite after acute renal injury induced by CLP. Furthermore, SES upraised IL-10 and mean arterial pressure and inhibited the levels of IL-1β and TNF-α. Further, this bioactive increased PPAR (peroxisome proliferator-activated receptor)-γ in the peritoneal macrophages and leucocytes in SIR induced by LPS. This group concluded that SES managed the macrophage and leucocyte PPAR-γ-linked systemic cytokines expression [35]. Wu and co-workers investigated the anti-inflammatory potential of SES in macrophages stimulated by LPS. This bioactive suppressed the prostaglandin E2, nitric oxide, and pro-inflammatory cytokine production. Furthermore, it markedly decreased protein and mRNA expression of COX-2 and iNOS. SES potentiated the antioxidant pathway signified by HO-1 and Nrf2. Additionally, SES inhibited the transport of NF-κB into the cell nucleus and reduced activation of MAPK, but it enhanced the activation AMPK (adenosine monophosphate-activated protein kinase). These facts advocate that this bioactive have an effect on oxidative and inflammatory damage [36]. In another study, Liu and the research team investigated the inhibitory effects of sesamol on systemic amyloidogenesis inflammation and neuroinflammation along with memory impairment. SES suppressed glial overactivation by inhibiting MAPK and NF-κB pathways along with inflammatory mediators’ (IL-1β and TNF-α) expressions [37]. In the year 2017, Yashaswini et al. investigated the effect of SES on LOX (inflammatory oxygenase-lipoxygenase) activity. It prevented the activity of soy LOX-1 (dose-dependent manner) with IC_50_ 51.84 μM. Additionally, sesamol inhibited the transformation of deactivated LOX (Fe_2_^+^) to active LOX (Fe_3_^+^) by preventing oxidation of iron and delaying the lag phase via scavenging hydroperoxides [38].

### 3.1. Anti-Cancer

Cancer is a life-threatening disorder for the human species nowadays. A commonly used treatment modality, chemotherapy is accompanied by various unendurable side effects. On the other hand, plants and their products are safer, low-cost, simple, less toxic, and eco-friendly in comparison to conventional treatment modalities [39]. A huge number of research studies have proposed that vegetable- and fruit-rich diets are associated with a lesser incidence of this dreadful disease, as these diets possess various groups of polyphenolic components with efficient anti-cancer activity. These polyphenolic components act as anti-cancer drugs via stimulation of cell cycle arrest, apoptosis, inhibition of GFR-mediated reaction, downregulation of protein kinase, and inhibition of NF-kB activation [40]. Thus, sesamol plays a vital role in improving chemo/radiosensitization and, hence, can act as a cancer management modality to provide a required dose at the tumor target site. various scientists showed how skillfully SES suppressed tumor cells’ metastasis and their growth potential [10].

Jacklin and his research team used endothelial cells for cell cycle arrest analysis. This team observed that at a large dose, sesamol accumulated in the S phase and transited via G2M was efficiently blocked [41]. In the year 2006, another group demonstrated the effect of SES on cytotoxicity induced by radiation in Swiss mice. Results of this group showed that this bioactive was efficient in spleen index maintenance, and enhancement in the elevation of endogenous spleen colony-forming units was seen. SES pretreated (50 mg/kg BW) Swiss mice proficiently suppressed inflammatory, mitotic, dead, and goblet cells. This bioactive also improved crypt cells, retained villus height, and stopped mucosal cell erosion. Nuclear enlargement in cells (epithelial) was found to be lower in sesamol-treated mice as compared to the irradiation control group. The radiation-induced reduction in endogenous antioxidant enzymes (GST, GSH, catalase) and the rise in lipid peroxidation have also been found to decrease by pretreatment with sesamol (50 and 100 mg/kg BW) at all post-irradiation time intervals [42]. In another experiment, it was found that sesamol pretreatment considerably reduced cytotoxicity, lipid peroxidation, and intracellular reactive species. In this study, DNA impairment and apoptotic morphological alterations in SES-pretreated and UVB-irradiated cells (HDFa) were investigated. Enhanced antioxidants status (enzymatic and non-enzymatic) was observed in sesamol plus UVB-irradiated HDFa cells [43]. Liu et al. investigated the relationship between SES and DNA by using UV radiations, circular dichroism, fluorescence, FT-IR, and molecular modeling. This team demonstrated that the fluorescence-quenching pathway of SES via ctDNA is through static quenching and saw that hydrogen bonding and lipophilic interaction might play a vital part in this interaction. Results of the cytotoxicity evaluation method showed that sesamol suppressed the proliferation of cancer cells; the apoptotic assay demonstrated that SES efficiently caused HepG2 cells’ death, and the nuclear localization experiment proposed that SES can move into and gather in the cell nuclei. These outcomes together validated that the anti-cancer potential of SES may be due to its interaction with DNA [44]. In the following year, Shimizu and co-workers demonstrated that SES retarded basal cyclooxygenase-2 (COX-2) transcriptional activities in the cancer cell line of the human colon (DLD-1). In male *Min* mice (6 weeks old), it was seen that SES treatment reduced the polyps number in the mid-portion of the small intestine. Moreover, this bioactive also inhibited cytosolic prostaglandin E2 and activity in the polyp portion. The outcomes of this experiment showed that SES may be act as a useful bioactive for cancer chemopreventives by suppressing COX-2 expression [45]. In the year 2016, the effect of SES on oxidative stress in colorectal HCT116 cancer cells was studied. The research team found that SES scavenged O_2_^−^ and DPPH and reduced the FRAP reagent. SES also decreased cell viability by interfering with cell cycle progression. Hence, it initiated the arrest of the S-phase and caused cell death via mitochondrial dysfunction, generating intracellular O_2_−, and DNA fragmentation in HCT116 cells [46]. In the same year, Siriwarin and Weerapreeyakul investigated sesamol’s anti-cancer potential and its mechanisms for apoptosis activity in SK-LU-1 (human lung adenocarcinoma) cells, specifically the biochemical and morphological changes (caspase 3/7 and apoptotic bodies activity, respectively). Cell lines demonstrated cell death caused by this bioactive. Enhanced caspase 8, and 9 activities exhibited that apoptosis has been induced by SES via both intrinsic and extrinsic pathways, respectively [47]. Another group of researchers explored the anti-cancer potential of SES and unveiled the significance of mitochondria in the anti-cancer potential of SES against HepG2 cells. Results of this group found that SES suppressed the formation of the cell colony, promoted arrest of the S phase during the progression of the cell cycle, and induced both extrinsic and intrinsic pathways of apoptosis (in vitro) in a dose-dependent pattern. Additionally, SES treatment prompted the dysfunction of mitochondria via impairing the mitochondrial membrane functions. Dysfunctioning mitochondria and increased production of H_2_O_2_ resulted in a disturbance of redox-sensitive signals [48]. In the same year, Bhardwaj and his research team found that SES reduced the burden of cancer and the level of lipid peroxidation, therefore decreasing the promotion and development of skin cancer. Further, a decrease in levels of bcl-2 and enhancement in the expressions of bax protein by this bioactive caused apoptosis in cancerous cells [49]. In the year 2019, Ma et al. studied sesame that prevented proliferation via JAK/STAT-3 pathway modulation in FTC-133 (thyroid cancer cell lines). Their outcomes revealed that sesame causes cytotoxicity and ROS production, which led to cell apoptosis in a time-dependent manner. In this investigation, it was observed that SES deactivated the translocation of STAT-3 and thus increased theBcl-2 and cyclin-D1 expression, and diminished caspase-9, 3 and Bax expression in chosen cell lines. Finally, it hindered expressions of thyroid cell and caused apoptosis by hindering translocation of STAT-3 [50]. Recently, the anti-tumor effect of sesamol in the Solid Ehrlich Carcinoma (SEC) model was confirmed, and enhancement of the anti-cancer potential of doxorubicin by SES was assessed [51]. Xiong and his research team explored the chemosensitizing efficacy of this bioactive against cervical cancer cells (HeLa). This group found improvement in paclitaxel activity by enhancing its toxicity against cervical cancer cells by ROS production, DNA damage, and decreased cell proliferation [52].

### 3.2. Cardioprotective

Cardiovascular diseases (CVD) represent the foremost reason for death globally, and the number of deaths has elevated at a fast rate regardless of the many innovations in the medical field. CVD terms include a collection of ailments related to the heart and blood vessels, in addition to hypertension, stroke (cerebrovascular disease), heart attack (coronary heart disease), heart failure, and peripheral vascular disorders. Herbal products, a well-known storehouse of various chemicals, have traditionally been an endless source of drugs. In recent years, significant interest has been shown in herbal or other natural products for the management of cardiovascular diseases. Numerous herbal plants have been precisely evaluated and found to be effective in the management of CVDs in recent years [53]. Sesamol and its derivatives are valuable, nutritious food-based polyphenols that demonstrate effective cardioprotective action. Being a powerful antioxidant, SES prevents oxidation of lipoproteins (low density), decreases aggregation of platelets, stimulates vasodilation, and improves the activity of endothelial nitric oxide synthase. Collectively, these events help in the prevention of atherosclerotic changes and represent an overall good cardioprotective potential. Intravascular thrombosis is the initiator of a wide range of cardiovascular disorders such as aggregation and adherence of platelets. Therefore, Chang et al. examined the in-depth mechanism of action of SES in inhibiting platelet activation in vivo and in vitro. This group showed that prevention of platelet aggregation is triggered by collagen via thromboxane A2 (TxA2) formation, [Ca_2_^+^] ion mobilization, and phospholipase C (PLC)γ2, MAPK phosphorylation and expression of protein kinase C (PKC). This study demonstrated that SES exhibits potent antiplatelet action, which comprises the initiation of the cAMP-eNOS/NO-cGMP pathway, suppression of the PLCγ2-PKC-p38 MAPK-TxA2 cascade, and, lastly, inhibition of aggregation of platelets [54]. The same group investigated the inhibition of the potential of SES in the NF-κB-mediated platelet function. This team concluded that SES triggers cAMP-PKA signaling, and then suppression of the NF-κB-PLC-PKC cascade, as a result of inhibition of Ca_2_^+^ ion mobilization and platelet aggregation [55]. Periasamy and the research team investigated the therapeutic potential of SES against isoproterenol-induced myocardial injury and infarction. Their experiments proved that SES effectively inhibited myocardial infarction by regulating proteolytic action and the expression of MMP-2 and -9 in isoproterenol-treated rats. The results displayed that blood pressure was lower, and heart rate, CKMB, ECG wave durations, myocardial injury, LDH, MMP-2, and MM-9 levels were enhanced in the SES-treated rats in comparison to the control [56]. In the same year, Vennila and Pugalendi investigated the effects of sesamol on tissue and plasma lipid profiles in isoproterenol (ISO)-induced myocardial infarction in male albino rats. SES saline solution was intraperitoneally administered once a day in the morning (for 7 days) and higher levels of total cholesterol, triglycerides, phospholipids, and free fatty acids in plasma and reduced tissue phospholipids levels were detected. These findings suggested that SES has anti-hyperlipidemic activity against cardiotoxicity [57]. In the year 2013, another research team evaluated the cardioprotective action of SES against cardiomyopathy induced by doxorubicin in rats. Results of this investigation exhibit that SES has cardioprotective action by normalization of doxorubicin-induced-altered biochemical factors such as troponin T, AST, LDH, and CK, and lipid profiles including triglycerides, cholesterol, LDL, HDL, and VLDL. It was concluded that SES has noteworthy cardioprotective action against cardiomyopathy through the amelioration of oxidative stress, membrane stabilization, and lipid-lowering effect [58]. In the same year, Hemalatha and co-workers investigated the antioxidant and antihypertensive potential of SES on DOCA (uninephrectomized deoxycorticosterone acetate)-induced hypertension in rats. In this experiment, levels of antioxidant enzymes such as superoxide dismutase, glutathione peroxidase, and catalase, and non-enzymatic antioxidants (vitamin E, vitamin C, and reduced glutathione) were estimated in plasma, erythrocytes, and tissues. Post-oral SES administration (50 mg/kg BW dose) remarkably reduced diastolic and systolic blood pressure, activities of hepatic marker enzyme, and products of lipid peroxidation and also increased the antioxidant potential [59]. Later on, Chen et al. examined the effects of SES on L5 levels of plasma, development of atherosclerosis in a rodent model, and L5-induced apoptosis in endothelial cells. This mechanistic research showed that this bioactive suppressed the L5-induced lectin-like oxidized LDL receptor-1 (LOX-1)-dependent phosphorylation of p38 MAPK and activation of caspase-3 activation and augmented eNOS and Akt phosphorylation [60].

### 3.3. Neuroprotective

It has been predicted that almost 1.5 billion humans throughout the world are suffering from central nervous system (CNS) ailments. The most burdensome of the CNS disorders are neurodegenerative disorders, which lead to age-related slow degeneration in neurological functions, frequently causing neuronal death. It has been revealed that different mechanisms such as neuroinflammation, protein aggregation, and oxidative stress are included in neuronal damage and death. Recently, the use of herbal moieties such as sesamol has been offered as an effective and alternative strategy in the management of neurological and neurodegenerative disorders [61]. VanGilder et al. administered SES to diabetic rats [induced by streptozotocin (STZ)] to study the role of oxidative stress on blood–brain barrier function and structure. This group measured structural and functional alterations in the blood–brain barrier by in situ brain perfusion and western blot analysis. The results demonstrated that SES treatment decreased the negative impact of streptozotocin-induced diabetes. Rats treated with SES showed a better antioxidant effect, which led to decreased expression of peroxynitrite, superoxide, and lipid peroxidation [62]. Kumar and his research team studied the protective action of SES against cognitive dysfunction (induced by 3-Nitropropionic acid), which reduced memory performance and was checked in the Morris water maze and raised plus maze. In contrast, 3-NP considerably induced oxidative stress and decrease total glutathione, reduced glutathione-S-transferase, glutathione, lactate dehydrogenase enzyme levels, and redox ratio in the striatum, hippocampal, and cortex parts, which are treated by SES pretreatment. This may be owing to its radical scavenging action [63]. Misra and his research group studied the effect of SES on Alzheimer’s disorder, employing the intracerebroventricular (ICV) streptozotocin-induced-cognitive impairment experimental paradigm. It was revealed that significant improvement in cognitive impairment, decreased action of acetylcholinesterase action, levels of TNF-α, and reduced oxidative–nitrergic stress in the brains of ICV streptozotocin-treated rats. Their findings demonstrated the efficiency of SES in inhibiting intracerebroventricular streptozotocin-induced cognitive impairment by modifying nitrergic signaling and oxido-inflammatory cascade [64]. In the year 2014, Hassanzadeh et al. evaluated the effect of SES in epilepsy, basically caused by cognitive impairment and oxidative stress. After treatment of male Wistar rats with pentylenetetrazole, kindling was developed, which was linked with myoclonic jerks and generalized tonic–clonic seizures, and also oxidative stress and cognitive impairment developed. SES significantly delayed kindling development and inhibited impairment induced by seizure and oxidative stress [65]. Hong and his research team evaluated the SES effect on infarcts and its potential to improve rats’ function with transient MCAO (middle cerebral artery occlusion). Functional efficacy was evaluated by employing a modified sticky-tape test (MST). It was found in this investigation that the activity of the hind limb in the SES group was significantly improved compared to the MCAO group during the whole experiment. These results demonstrated this bioactive induced functional improvement and could be beneficial for the treatment and management of ischemic stroke [66]. Later on, in 2018, Ren and co-workers investigated the anti-oxidative and neuroprotective potential of SES on memory impairments and neuron damage induced by oxidative stress. For their study, firstly, mice (C57BL/6J) were treated with D-galactose intraperitoneal injections for 8 weeks. Behavioral tests, including the water maze-test and Y-maze test, demonstrated that SES significantly improved cognitive impairments. Meanwhile, this bioactive improved neuronal damage and enhanced BDNF levels in the rat hippocampus. SES enhanced levels of mRNA and protein expressions of antioxidant enzymes NQO1 and HO-1along with reduced inflammatory cytokines IL-1β and TNF-α in mice treated with D-galactose. Further, the activity of GSH and CAT levels was found to increase in SES-treated mice serum [67].

### 3.4. Hepatoprotection

Hepatic disorders are the primary cause of morbidity and mortality worldwide. These disorders are provoked by obesity, unhealthy lifestyles, and alcohol and drug consumption. The employment of medicinal plants in the treatment of liver disorders has been described since ancient times. In the case of liver disorders, various herbal medicines have been shown to ameliorate hepatic impairments [68].

Sesamol was examined for its effects on mortality and liver injury associated with reactive oxygen species in Wistar rats. By calculating the levels of hepatic hydroxyl radicals, lipid peroxidation, nitric oxide production, and superoxide anion generation, liver disorders can be evaluated. It was observed that hepatic hydroxyl radicals, superoxide anion, and lipid peroxidation levels were found considerably declined in SES-treated septic animals [69]. In the following year, the same group of researchers investigated the effect of SES on hepatic function and oxidative stress (systemic) in mice with acute iron poisoning. Sesamol declined levels of hydroxyl radicals, superoxide anion generation, lipid peroxidation, xanthine oxidase potential, and iron production in mice with iron poisoning. Additionally, this also declined the alanine aminotransferase and aspartate aminotransferase levels in serum and alleviated iron-poisoning-induced histological variations in the liver [70]. Periasamy et al. examined the therapeutic efficacy of SES in rats with monocrotaline-induced sinusoidal obstruction syndrome (SOS). Many parameters viz. alanine transaminase, aspartate transaminase, Kupffer cells, mast cells, myeloperoxidase, neutrophils, tissue inhibitor of matrix metalloproteinase-1 (TIMP-1), matrix metalloproteinase-9 (MMP-9), collagen, and laminin were investigated. All the above-mentioned parameters, except for collagen, laminin, and TIMP-1, were found to be potentially lesser in SES-treated animals in comparison to monocrotaline-treated animals. It was concluded from this investigation that a single therapeutic dose of SES reduced SOS via diminishing the generation of inflammatory cells, upregulating TIMP-1 expression, and downregulating MMP-9 [56].

In the year 2014, Jnaneshwari et al. evaluated oxidative damage and organ toxicity induced by cyclophosphamide (CP) and their alleviation using SES. Increased levels of lipid peroxidation and endogenous ROS, with declined levels of total thiols, and glutathione, as well as diminution in antioxidant enzymes (catalase, superoxide dismutase, glutathione peroxidase, and glutathione transferase), were noticeably found in CP-treated animal models. Pro-inflammatory mediators such as IL-1β, TNF-α, cyclooxygenase-2, and IL-6 were also reported to increase. Furthermore, the hepatic function markers such as serum aspartate alanine aminotransferase and alanine aminotransferase levels in serum were found to change. Changes in these parameters were potentially reinstated to usual levels after using SES (50 mg/kg) via the oral route. These findings indicated its considerable anti-inflammatory, hepatoprotective, and antioxidative stress capabilities [71].

### 3.5. Wound Healing

Wound healing and tissue repairing are complex procedures, in which inflammation, granulation, and tissue remodeling are involved [72]. The wound initiates a complex chain of cascades that imply interactions of numerous cells, growth factors, cytokines, extra-cellular matrix proteins (ECM), and their mediators. In India, topical use of herbal drugs is a common home treatment for various health issues such as insect bites, chicken pox, and skin diseases. Based on the ancient employment of herbal drugs in the healing of wounds, some investigations by research scientists assessed the role of SES in fastening wound healing [73].

Kiran and Asad assessed the influence of sesame seeds and their oil on induced incision wounds, excision wounds, dead space wounds, and burn wound models in experimental animals (rats). The treated rats exhibited a potential decrease in the period of epithelization and contraction of wounds (50%). In the case of the incision wound model, a potential augmentation in the breaking strength was also detected. In the dead space wound model, oil and seeds treatment significantly increased the dry weight, breaking strength, and hydroxyproline amount in the granulation tissues. The findings suggested that sesame seeds and their oil administered orally or applied topically have promising wound healing potential [74].

In the year 2011, Shenoy and his research team investigated the effects of SES on wound healing in albino rats (both in dexamethasone (DM) delayed and normal processes of healing). The findings presented that tensile strength has been potentially (*p* < 0.05) augmented with SES treatment in comparison to control in both cases (DM suppressed healing and normal healing). Moreover, SES-treated animals presented a remarkable increase in hydroxyproline contents in comparison to control. These outcomes ascertained the promising nature of this bioactive in delayed and normal wound healing [75].

### 3.6. Anti-Aging

Aging occurs due to extrinsic factors such as smoking, pollution, sun exposure, and diet or intrinsic factors such as cellular metabolism, metabolic process genetics, and hormones. In this era of modern health science, people select natural herbs instead of laser therapy or plastic surgery to handle aging complications and look younger as well. Herbs supply nutrients necessary for healthy skin and further aid in skin functioning. These possess numerous phytoconstituents such as terpenoids, polyphenols, and carotenoids, which show anti-aging potential [76]. Sharma and Kaur investigated the anti-aging potential of SES. Outcomes from histopathological and biochemical studies ascertained that SES formulation was found efficacious in preventing photo damage (ulcers, changes in skin integrity, and lesions) from chronic exposure to UV. This investigation presented the promising therapeutic efficacy of SES formulation (having antioxidant potential) in handling UV-induced harm caused to skin tissues (made up of elastin and collagen) [77].

In the year 2012, another constituent of sesame oil, i.e., sesamin, has also been evaluated for its anti-aging potential in the D-galactose-induced senescent mice model. It was observed that the changes in motor and memory ability, body weight, glutathione peroxidase (GSHpx), liver superoxide dismutase (SOD), malondialdehyde (MDA), and reduced glutathione (GSH) took place. Sesamin treatment potentially enhanced the hepatic GSHpx, SOD activities, body weight, proliferative response, GSH content, and IL-2 splenocyte generation induced by ConA. Further, this treatment remarkably declined hepatic macrophage proliferative response levels, MDA content, and NO and IL-1generation. From the findings, it was noted that sesamin possesses a promising anti-aging activity in D-galactose-induced senescent mice [78]. Recently, lipoic acid–sesamol conjugate has been prepared and evaluated for its anti-aging parameters against *Drosophila melanogaster* (*Canton-S).* This group observed that IC_50_ 30 µΜ increases the life span of female flies up to 33 days i.e., 18% compared to the control group [79]. Hence, the above experiments opened a new window to use this bioactive as an anti-aging agent.

### 3.7. Anti-Candidal

Over the last few years, there is a large increase in the incidences of Candida infections owing to the growing body of the immunocompromised population. Currently, undesirable toxic side effects and the appearance of resistant strains to the available antifungal agents constitute noticeable clinical issues for candidiasis management. Thus, molecules isolated from natural sources showing potential antifungal efficacy are a hopeful source for the development of novel anti-candidal delivery systems. The phenolic compounds derived from natural sources that possess antifungal properties are potential alternatives [80]. Ansari et al. investigated the antifungal effects of SES via disruption of a calcineurin signaling pathway in *C. Albicans* (a human fungal pathogen). SES treated *C. Albicans* was observed to copy the phenotype exhibited by cells. Hence, a fault in the calcineurin signaling pathway leading to sensitivity against ionic, salinity, alkaline pH, membrane, serum, and endoplasmic reticulum stresses was also seen. The findings of this investigation recognized the antifungal property of SES that can be utilized for the management of candidal infections [81]. In the year 2016, the same research group investigated the mechanism of the anti-candidal effect of sesamol. The inhibition of significant virulence in the fungus was attributed due to biofilm formation, morphological transition, and epithelial cell adhesion. The amendment in iron homeostasis as discovered by ferroxidase assay for estimation of iron levels, hypersensitivity under iron deprivation, and associated upregulation of FTR2 was also presented in this study. It was concluded that this bioactive was found to be responsible for mitochondrial dysfunctioning and DNA alterations [82].

### 3.8. Anti-Ulcer

Ulcers are inflammations or breaks in the mucus membrane or skin lining in the alimentary tract. Their progression happens as a result of interruption in the normal balance due to decreased mucosal resistance or augmented aggression. The gastric mucus membrane is always exposed to potentially harmful agents such as pepsin, acids, food ingredients, bile acids, drugs, and bacterial products (*Helicobacter pylori*). These agents have been directly associated with the pathology of gastric ulcers. Additionally, increased pepsin and gastric acid secretion, cell proliferation growth, hampered prostaglandin production, and decreased gastric motility and gastric blood flow play a significant role in ulcers. Numerous herbs and spices have been investigated by the research community for their anti-ulcer potential; encouraging results have been attained [83]. Hsu et al. evaluated the defensive potential of SES in acute gastric-mucosal-damaged rats (induced by diclofenac). It was observed that SES declined hydroxyl radical levels and lipid peroxidation in the mucosa, and it potentially controlled decreased glutathione levels and reduced diclofenac-induced prostaglandin E2 production and COX [84]. Furthermore, in the following year, the same research group evaluated the influence of SES plus aspirin on gastric mucus membranes in rats. The findings showed decreased gastric hemorrhage, and mucosal ulceration (induced by aspirin) and potentially declined nitric oxide production, myeloperoxidase activity, lipid peroxidation, and pro-inflammatory cytokines in gastric mucosa concerning the aspirin alone group in a dose-dependent manner. It was hypothesized that SES plus aspirin diminished gastro toxicity (by preventing neutrophil infiltration and following gastric mucosal oxidative stress and inflammation) induced by aspirin in rats [85]. Hsu and his research team again investigated the role of this bioactive on water immersion restraint-induced stress-associated mucosal disorder in rats. The treatment of SES potentially declined gastric hemorrhage and ulceration. The inhibition of mucosal IL-1β, IL-6, and TNF-𝛼generation and NF-𝜅B potential of SES was also observed in water immersion restraint-treated animals. Additionally, augmented CD68 and myeloperoxidase levels in the gastric mucus membrane were observed in water immersion restraint-treated rats [86]. Later on, Sori et al., evaluated the anti-ulcer potential of the seed extract of sesame in rat animal models (stress-induced peptic ulcers). The extract remarkably diminished mucin content, gastric lesions, gastric pH, the volume of gastric juice, and total and free acidity concerning positive control animals at both low and high doses. The sesame extract (at high doses)-treated animals presented comparable outcomes such as influence on pH, gastric volume, mucin content, total acidity, and free acidity with standard treatment animals [87].

## 4. Formulation Strategies for Sesamol

**Figure 4:**
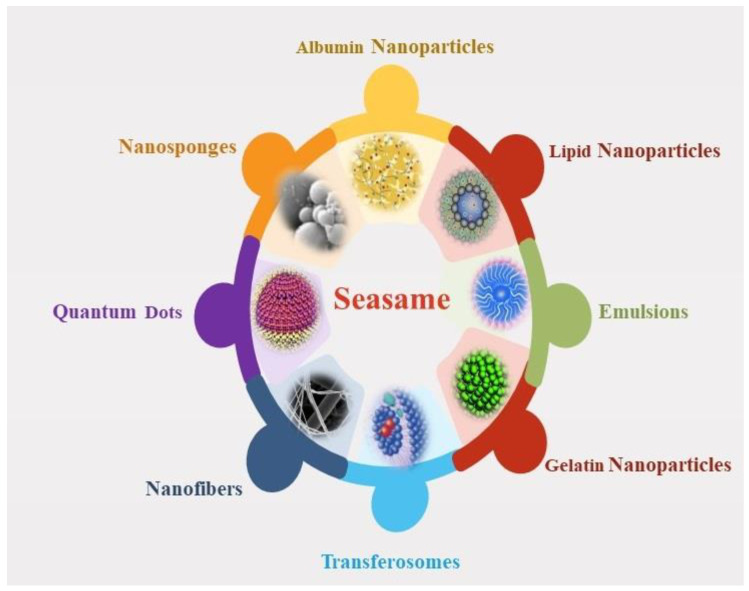
Nanoformulations of Sesamol.

Several formulation approaches investigated for SES delivery have been explored, which are discussed in the following subsections and systematically categorized in Table 1. A limited number of formulation investigations were performed for SES (Figure 4), which have been focused to minimize the issues associated with bioavailability, stability, and solubility augmentation of this bioactive. These challenges compromised the safety and efficacy of SES.

### 4.1. Lipid-Based Delivery Systems

#### 4.1.1. Solid Lipid Nanoparticles (SLNs)

SLNs are colloidal drug delivery systems consisting of lipid that remains in solid form (both at body and room temperatures). These include the benefits of liposomes, emulsions, and polymeric nanoparticles while reducing their drawbacks. This system mainly comprises a solid matrix core (hydrophobic) which is coated with a phospholipid. The tail regions (hydrophobic) of the phospholipid are implanted into the core. SLNs are fabricated by employing solid lipids (e.g., fatty acids, purified triglycerides, waxes, or steroids), water, and emulsifiers [103]. The merits of this delivery system include its lipid materials, which are both biocompatible and biodegradable, controlled release pattern, protection of encapsulated moieties, and improved solubility. The use of this lipid carrier to improve the bioavailability and bioactivity of various bioactive including sesamol has been reported in recent times [18]. In this setting, Kakkar and Kaur tried to load sesamol into solid lipid nanoparticles (SES-SLNs) to minimize its distribution to tissues and achieve its targeting of the brain. They prepared three scale-up batches of SES-SLNs via micro emulsification technique that were found to be stable for three months at 5 ± 3 °C [89]. Geetha and co-workers incorporated sesamol into SLN to enhance its bioavailability in the skin for skin cancer. The topical application of drug-loaded SLN was shown to be the most efficacious system and in vitro anti-proliferative (employing MTT assay) and DNA fragmentation activity in HL 60 and MOLT-4 cell lines, authenticated the apoptotic behavior of SES [91]. In another study, sesamol was packed into SLN and explored the hepatoprotective activity of SES-SLNs when compared to free drugs and established SES-SLNs as a therapeutic choice for CCL4-induced hepatotoxicity [93].

#### 4.1.2. Nanostructured Lipid Carriers (NLCs)

NLCs are the second-generation lipid carriers generated to circumvent tissues allied with SLNs (Solid Lipid Nanoparticles) and are used in numerous therapeutic applications [104,105]. NLCs are carrier systems prepared by both liquid and solid lipids. Glyceryl behenate, triglycerides, glyceryl palmito stearate, steroids, fatty acids, and waxes are prevalent solids cast in NLCs and oils typically are digestible oils obtained from a natural origin [106]. The biocompatible characteristic of lipids is behind their preparation as efficient drug delivery for various drugs including SES.

In 2016, Puglia and the research team developed two different NLC systems dependent on a similar solid lipid (Compritol^®^ 888 ATO, Gattefossé, Lyon, France), but in a mixture with two varying types of oil phases such as sesame oil (nanostructured lipid carrier-PLUS) and Miglyol^®^ 812 (nanostructured lipid carrier-M). In vitro evidence showed that NLC dispersions were capable to conduct the rate of SES diffusion across the skin, concerning the reference formulations and extended antioxidant study of this moiety, especially when delivered by NLC carrier-PLUS was confirmed [94]. In the following year, another research group formulated SES-NLCs and evaluated the bioactivity of the selected formulation in global cerebral ischemia/reperfusion (I/R) injury and oxygen–glucose deprivation (OGD) and the involvement of the phosphatidylinositol 3-kinase (PI3K) pathway. They have concluded that SES-NLCs enhanced the pharmacological activity of SES and supply prolonged protective results. This carrier system, by activating the PI3K pathway, may act as an efficacious candidate for neuroprotection in the case of cerebral stroke or another neurodegenerative disease [95].

### 4.2. Emulsion-Based Delivery Systems

#### 4.2.1. Emulsion

Emulsion can be explained as a biphasic system comprising two immiscible liquids: first, the dispersed phase is uniformly and finely suspended as globules across the emulsion, and the second is the continuous phase. However, emulsions are a thermal-sensitive system, and so a third substance, the emulsifier, is used to stabilize the system. There are two common types of emulsions: water in oil (W/O) and oil in water (O/W). Emulsions have various benefits over other formulations, as the solubilized drug has more bioavailability. Furthermore, gastrointestinal issues and first-pass metabolic effects are also circumvented [107]. Alencar et al. developed dry SES emulsions from different combinations of saccharose with sodium caseinate (SC) or hydroxypropylmethylcellulose (HPMC), employing spray-drying or freeze-drying methods. Emulsions were analyzed for their antioxidant activity in cultured 3T3 murine fibroblasts and in an ex vivo model of UV-irradiated rat skin. They observed that the overall antioxidant behavior of reconstituted emulsion was improved. They also believe their method possesses a very economical alternative to oils for the promising pharmacological or topical application of these bioactives [88]. In 2018, another research team fabricated biopolymer conjugates from hyaluronic acid (HA) and lactoferrin (LF) and investigated their efficacy as emulsifiers for preparing SES-embedded emulsions, and a stability study was performed. They showed that the conjugates enhanced both the chemical and physical stability of the emulsions over storage and also suggest that LF-HA conjugates can be efficacious emulsifiers for application in foodstuff and other uses [97].

#### 4.2.2. Microemulsion-Based Hydrogel

Microemulsions are stable under thermal conditions and isotropically transparent dispersion of two immiscible liquids, such as water and oil, stabilized using an interfacial film of surfactants, having a size of 5–200 nm, and consisting of quite a bit less interfacial tension. Due to their specific solubilization characteristic, microemulsions have lured keen attention as a promising drug delivery system, either as bioavailability enhancers or as carriers for topical delivery of hydrophobic molecules. The benefits of microemulsions involve their optical clarity, thermodynamic stability, ease of preparation, and higher absorption and diffusion rates in comparison to solvents with no surfactant. This issue may be circumvented by preparing microemulsion-based hydrogel employing polymers such as carbopol, hydroxy propyl methyl cellulose (HPMC), and xanthan gum [108]. Recently, Ghorbanzadeh and his research team prepared microemulsion-based hydrogel of sesame oil, and formulations were characterized for zeta potential, particle size, refractive index, polydispersity index, electrical conductivity, stability, and pH value. The clinical studies of formulation on the skin of guinea pigs involved skin irregularity, scaling, hyperpigmentation, erythema, and edema. Epidermal, hyperpigmentation, hyperkeratosis, acanthosis, exocytosis, chromatin discoloration in the nucleus of epidermal squamous cells, dermal vascular hyperemia, edema, perifolliculitis, and skin thickness, infiltration of plasma cell eosinophils, and lymphocytes into dermis were analyzed by histopathological analysis. Their results demonstrated that this carrier system has the potential for UV protection, and its topical delivery may reduce the dermal side effects due to sun rays [98].

### 4.3. Vesicular Delivery Systems

#### 4.3.1. Micelles

Micelles are promising drug delivery carriers for poorly soluble and bioavailable compounds by virtue of their unique core–shell architecture. The inner hydrophobic core allows the encapsulation of hydrophobic drugs, thus enhancing their bioavailability and stability. The choice of core-forming polymers is the main determinant for the characteristics of polymeric micelles (PMs) such as drug loading efficacy, release profiles, and stability. Poly(propylene oxide) (PPO) such as hydrophobic poly (amino acids), poly (lactic acid) (PLA), copolymers of lactic acid and poly (caprolactone) (PCL), and glycolic acids are considered to be generally used core-forming blocks of PMs and poly (ethylene glycol) (PEG) is frequently employed as the hydrophilic parts of the block copolymers; however, it is a biocompatible polymer with approval of FDA as an ingredient of numerous pharmaceutical formulations [109]. In 2017, Yashaswini and co-workers improved SES bioavailability by encapsulating it in mixed phosphatidylcholine micelles. SES in phosphatidylcholine mixed micelles exhibited increased bioavailability together with enhanced anti-inflammatory effects. The anti-inflammatory activity of free SES and loaded were compared by employing an LPS-treated RAW 264.7 cell line and lipoxygenase inhibition. Phosphatidylcholine mixed micelles (PCS) affected the downregulation of NO production, iNOS protein expression, lipoxygenase, and ROS restriction as compared to free SES [19].

#### 4.3.2. Transfersomes

Transfersomes are defined as a novel drug delivery system in which specially fabricated vesicular particles reside in at least one inner aqueous compartment surrounded by lipid vesicles; these are liposomes in morphology, but, by function, are suitably deformable to move via pores much lesser in comparison to their size. These consist of edge activators, ethanol, phospholipids, and sodium cholate and are employed in a non-occlusive manner [110]. Transfersomal formulations are decidedly employed in transdermal immunization and peripheral drug targeting, as well as well-recognized to be a foremost delivery system for the transdermal delivery of a massive array of therapeutic moieties. Over a couple of decades, the utilization of the transfersomes in the arena of various herbal drugs including epigallocatechin-3-gallate, resveratrol, paclitaxel, curcumin, etc., have been studied [111]. Recently, fluconazole-loaded sesame oil enclosing nanotransfersomes fabricated by the thin-layer evaporation method has been studied to enhance the local treatment of oral candidiasis. Their results showed that value-added antifungal effectiveness in hyaluronic acid-based hydrogel may be by its synergistic action of sesame oil, hyaluronic acid, and fluconazole [101].

### 4.4. Miscellaneous Delivery Systems

#### 4.4.1. Gelatin Nanoparticles (GNPs)

Gelatin is a protein, produced from the hydrolysis of collagen. Gelatin is an appealing biodegradable substance for application in nano-pharmaceutics and nano-biotechnology. GNPs (gelatin nanoparticles) are biodegradable, inexpensive, and nontoxic and help in terms of drug delivery and sustained drug release. GNPs should consist of a high drug loading capacity to carry the desired quantity of the drug. These nanoparticles have been broadly used as gene and drug carriers to target diseased tissues involving tuberculosis, cancer, and HIV infection together with the cure of restenosis and vasospasm, due to their biodegradability and biocompatibility [112].

ElMasry and the research team successfully synthesized oleic acid chemically modified GNPs for SES transdermal delivery. They performed in vitro release of it from the newly fabricated system and compared it to the sesamol solution. Permeation was analyzed via albino mice skin employing a newly fabricated UPLC-MS/MS technique where SES-loaded GNPs showed better permeability. Further cytotoxicity studies on MCF-7 breast cancer cells demonstrated that these nanoparticles showed the least IC_50_ value (595 μM ± 32.3) [96].

#### 4.4.2. β-Cyclodextrin Inclusion Complex

Cyclodextrins (CDs) are physically and chemically stable macromolecules obtained via enzymatic degradation of starch. They are hydrophilic and non-toxic, having a lipophilic, cavity, and hydrophilic outer surface. The most frequent natural CDs are 𝛾, 𝛽 and 𝛼 with 8, 7, and 6 glucopyranose units, respectively [113]. From these, 𝛽-CD is ideal for complexation owing to its perfect cavity size, efficient drug loading and complexation, availability, and relatively inexpensive cost. Numerous hydrophobic, hydrophilic, and ionic derivatives have been prepared and used to enhance the biopharmaceutical and physicochemical characteristics of drugs and the inclusion capability of natural CDs. Randomly methylated-(RM-𝛽-CD), hydroxypropyl-(HP-𝛽-CD), and sulfobutyl ether-𝛽-cyclodextrin (SBE-𝛽-CD) are mainly considered for complexation. Owing to higher complexing and solubilizing properties, these are mostly preferred for complexation nowadays. CDs form inclusion complexes with a wide array of hydrophobic molecules and alter the biological and physicochemical effects of guest moieties [114].

Ma et al. prepared an SES and HP-β-CD complex to improve its water solubility. They have observed that the solubility of this drug was significantly enhanced via the inclusion complex. They also performed antioxidant activity which showed lower antioxidant activity of SES in an inclusion complex when compared with a pure drug, which was due to the high content of HP-β-CD [90].

#### 4.4.3. Floating Beads

Floating beads are low-density vehicles that have enough buoyancy to float upon the gastric contents and last in the abdomen for an extended period. Although the beads float on top of the gastric contents, the moiety is sustained and released at the expected rate, which exhibits improved gastric residence time (GRT) and decreased fluctuation in plasma drug concentration. The common approach for fabricating these systems includes resin beads embedded with bicarbonate and coated with ethyl cellulose. The coating, which is permeable but insoluble, permits water penetration. Hence, beads float in the stomach due to the release of carbon dioxide [115]. Geetha et al. first developed multiunit gastro-retentive floating beads (FBs) for localized and extended release of SES for treatment of gastric cancers, which were characterized and evaluated in vivo in N-methyl-N-nitro-N-nitroguanidine-induced gastric cancer in rats. Outcomes demonstrated that the drug in floating beads significantly decreased the release (diffusion controlled) rate, enhanced t50% (31 times), and decreased its in vivo clearance (41.5 times). The preclinical studies exhibited SES-FBs (10 mg/kg) to be more promising than free drugs [92].

#### 4.4.4. Nanofibers

Nanofibers are drug carriers with diameters in the nanometer range and due to their ultra-high surface area give potential benefits for drug delivery. In addition, nanofibers may be prepared of various biodegradable polymers and natural materials by sol-gel and electrospinning techniques. A broad range of moieties involving anti-cancer drugs, proteins, antibiotics, RNA, and DNA may be encapsulated in nanofiber scaffolds [116]. Recently, Liu and his research team incorporated SES into a cellulose acetate (CA)–zein composite nanofiber membrane and evaluated the wound-healing process for diabetic mice. The composite nanofiber membrane with a higher dose of SES (5% *w*/*w* polymer concentration) encouraged the preparation of myofibroblasts by increasing TGF-β signaling pathway transduction and elevated keratinocyte growth by hindering chronic inflammation in wounds, hence increasing the healing of wounds in diabetic mice. This work has widened the utilization range of SES and provided a reference for the design and fabrication of novel wound dressings in the future [100].

#### 4.4.5. Quantum Dots (QDs)

QDs are nanoparticles that have demonstrated great potential in applications of various biomedical and biological, especially in drug activation/delivery and cellular imaging. Structurally, QD consists of a metalloid crystalline core, which ultimately depends on its size and composition. The core consists of substances involving cadmium–tellurium (CdTe), cadmium–selenium (CdSe), indium arsenate (InAs), or indium phosphate (InP). Due to its small size, QD as a drug delivery carrier presents considerable benefits in the region of pharmacokinetic characteristics and efficient drug load in comparison to traditional systems. Further benefits include the alteration of their surface chemistry to suit the addition of targeting and therapeutic molecules for clinical uses [117]. In 2019, Abdelhamid et al. improved the cytotoxicity of SES by encapsulating it into cadmium sulfide (CdS) quantum dots (QDs) modified chitosan (CTS). The cytotoxic assay demonstrated that sesamol-CdS@CTS was more effectual against cancer cells in comparison to the drug solely. The findings exhibited that the IC_50_ values of CdS@CTS were 1730 ± 54, sesamol were 495 ± 16.4, and sesamol-CdS@CTS were 117 ± 3.2 μg/mL [99].

#### 4.4.6. Nanosponges

Nanosponges are novel hyper reticulated systems fabricated by cross-linking cyclodextrin polymer with various organic bridging agents leading to the construction of nano-range cavities to encapsulate drug moieties. Typically, nanosponges have been constructed from cyclodextrin cross-linked with organic carbonates. Nanosponges mainly comprise three components: the polymer, cross-linking agent, and drug moiety. This nanosystem offers a flexible platform to overcome hindrances such as stability, solubility, toxicity, and bioavailability for synthetic and herbal moieties [118,119]. To date, numerous bioactives has been encapsulated in this nanosystem, including babchi oil [120], curcumin [121], azelaic acid [122], ellagic acid [123], and piperine [124]. Recently, sesamol-loaded nanosponges were fabricated for assessment of stability, antioxidant, and anti-tyrosinase potential. The findings suggested successful entrapment of this bioactive and enhancement of its stability of it. Further, anti-tyrosinase and antioxidant potential were also found preserved after sesamol was loaded into nanosponges [4]. Hence, this system is effective in preserving therapeutic activity and acts as a shield for bioactive stability issues.

#### 4.4.7. Albumin Nanoparticles

Albumin is an alluring delivery system owing to its advantages such as its abundance in plasma, biocompatibility, nonimmunogenicity, biodegradability, and safety profile. There are two techniques for albumin nanoparticle fabrication i.e., the chemical-based technique and the physical-based method [125]. Further, albumin protein has a remarkable capability to bind with lipophilic molecules, which include fatty acids and hormones [126]. In the recent past, Zaher and his research team formulated a sesamol-loaded albumin nanosystem to check cellular oxidation protection of free and albumin nanoparticle conjugated drugs. Moreover, sesamol stability and pharmacokinetics were enhanced. The research team also found enhanced efficacy and biochemical assessment of formulation against cardiac biomarkers, lipid peroxidation, and liver enzymes [102].

## 5. Toxicity of Sesamol

The National Cancer Institute (NCI) nominated sesamol for carcinogenicity testing with a moderate to high priority depending on the efficacy of extensive human exposure to SES. Positive test outcomes in the mouse lymphoma assay and deficiency of adequate chronic toxicity, carcinogenicity, and epidemiology data are accessible for this drug. However, no data were available on the reproductive, carcinogenic, mutagenic, teratogenic, or immunologic activities of SES in humans. Additionally, no data were found on the chemical disposition of this compound in humans. In mice, intraperitoneal LD_50_ for SES was recorded to be 470 mg/kg. This bioactive was observed to induce irritation and necrosis during intradermal injection in rabbits and rats, respectively. In rabbits, SES caused reversible ocular irritation when injected into the conjunctival sac. Chronic and pre-chronic feeding of high doses of SES showed weight loss and weight gain in the kidney and liver of rats and mice. This bioactive was not observed to have hepatocarcinogenic effects via oral route to rats in pre-chronic evaluations. SES tested positive with and without metabolic stimulation in the mouse L5178Y (Tk+/−) lymphoma activity. Moreover, SES was non-mutagenic in *Salmonella typhimurium* with and without metabolic activation. The basic moiety of SES, isosafrole, safrole, and dihydrosafrole is the same, i.e., methylene dioxyphenyl. The latter three molecules have been observed to be carcinogenic in mice and rats. Isosafrole and safrole build liver tumors in rats and mice. Safrole also produced lung tumors in infant mice, and dihydrosafrole generated esophagus tumors in rats, and lung and liver tumors in mice [127,128,129].

The structural phytonutrient and simple-type analogs of SES molecules that have been analyzed to date have relatively less direct toxicity in mammals. In rodents, the parent food source analog, benzodioxole, had a higher LD_50_ of approx. 0.5 g/kg and an extended period of exposure over are needed to cause any toxic effect. In humans, there is much less information is available regarding the toxicity of simple sesamol structures. However, a single dose of 50 mg of synthetic SES synergist piperonylbutoxide produces no side effects; this dose was guessed to be approximately 50-fold higher than the maximum daily dose. Furthermore, it is still an open question whether extended exposure to SES agents such as piperonylbutoxide produces direct harmful effects on humans. However, any bioactive that a person is constantly exposed to, even if it is from a plant-derived food source, may cause some extent of toxicity. In that line of argument, plants with SES food nutrients are related, to some extent, to toxicity, which can be acceptable at a threshold level of consumption in humans [129,130,131,132].

## 6. Conclusions

In the past couple of years, pharmaceutical formulation experts have witnessed a paradigm shift toward the development of herbal formulations that curtail numerous life-threatening disorders including cancer, cardiovascular dysfunctions, and neurodegenerative diseases. Similarly, sesame oil and particularly sesamol (the main constituent) give valuable contributions in circumventing and treating several health issues. However, this phenolic compound possesses low bioavailability, stability, and low water solubility, and accordingly, a higher dose is needed to achieve therapeutic levels. These attributes hamper the utility of this bioactive in clinical applications. Therefore, in this review bioactive as well as novel drug delivery strategies are covered in detail. Various carrier systems have been explored such as lipid nanoparticles, emulsions, polymeric nanoparticles, gelatin nanoparticles, and inorganic materials and protein-based carriers for delivery of SES. Among these, the majority of works have been focused on polymeric and lipid nanocarriers, perhaps owing to their biocompatibility. Further, the most noticeable therapeutic activities of sesamol, which include antioxidant, anti-inflammatory, and apoptosis through its regulatory influences on molecular targets, participated in the management of various chronic diseases. Hence, this review paves the way for further development of multifunctional formulations of SES in the nutraceutical and pharmaceutical industry in the future.

## Figures and Tables

**Table 1:** Novel Formulations of Sesamol.

Type of Formulations	Techniques	Methods	Results	References
Emulsion	Spray-Drying or Freeze-Drying Techniques	In vitro and in vivo (murine 3T3 fibroblasts, male Wistar rats)	Improved antioxidant activity Improved UV protection	[88]
Solid Lipid Nanoparticles	Micro-Emulsification Technique		Increased stability	[89]
Hydroxypropyl-β-Cyclodextrin	Freeze-Drying Technique		Improved in solubility	[90]
Solid Lipid Nanoparticles		In vitro and in vivo (Molt-4 and HL-60 Cell lines; Laca mice)	Improved in bioavailability Improved anti-cancer effect	[91]
Floating Beads	Orifice Ionic Gelation Technique	In vitro and in vivo (male Wistar rats)	Improved diffusion controlled release, enhanced t50% (31 times), decreased clearance 41.5 times), improved therapeutic action against gastric cancer	[92]
Solid Lipid Nanoparticles	Micro-Emulsification Technique	In vitro and in vivo (male Wistar rats)	Improved oral bioavailability, improved controlled release, reduced irritation and toxicity, improved hepatoprotection	[93]
Nanostructured Lipid Carriers	High Shear Homogenization with Ultrasound	In vitro (excised human skin membranes i.e., SCE)	Improved diffusion controlled release, improved antioxidant activity	[94]
Nanostructured Lipid Carriers	High-Pressure Homogenization	In vitro and in vivo (PC12 cells, Wistar rats)	Improved stability, improved controlled released, enhanced retention and efficiency, prolonged neuroprotective effect	[95]
Micelles	Solvent Evaporation Technique	In vitro (Caco-2 cells and RAW 264.7 cells)	Improved bioavailability, improved anti-inflammatory activity	[19]
Gelatin Nanoparticles	Desolvation Technique	In vitro (MCF-7 breast cancer cells)	Improved targeting by intracellular delivery, improved cytotoxic effect	[96]
Emulsion	High-Pressure Homogenization Technique		Improved stability	[97]
Microemulsion-Based Hydrogel		In vivo (guinea pig)	Improved stability, improved UV protection	[98]
CDs-Modified Chitosan Quantum Dots	Ultrasonication Technique	In vitro	Improved anti-cancer activity	[99]
Nanofibers		In vitro and in vivo (mice)	Promoted myofibroblasts formation, promoted keratinocyte growth, enhanced wound healing	[100]
Transfersomes	Thin-layer Evaporation Technique	In vitro	Enhanced anti-fungal activity	[101]
Nanosponges	Solvent Evaporation Technique	In vitro	Enhanced stability, preserved antioxidant and anti-tyrosinase activity	[4]
Albumin Nanoparticles	Desolvation Technique	In vitro and in vivo	Enhanced stability and protective effect of oxidative stress	[102]

## Data Availability

Data is contained within the article.
